# Melatonin ameliorates PM_2.5_‐induced cardiac perivascular fibrosis through regulating mitochondrial redox homeostasis

**DOI:** 10.1111/jpi.12686

**Published:** 2020-11-16

**Authors:** Jinjin Jiang, Shuang Liang, Jingyi Zhang, Zhou Du, Qing Xu, Junchao Duan, Zhiwei Sun

**Affiliations:** ^1^ Department of Toxicology and Sanitary Chemistry School of Public Health Capital Medical University Beijing China; ^2^ Beijing Key Laboratory of Environmental Toxicology Capital Medical University Beijing China; ^3^ Core Facilities for Electrophysiology Core Facilities Center Capital Medical University Beijing China

**Keywords:** fine particulate matter, melatonin, mitochondrial ROS, perivascular fibrosis, SIRT3, SOD2 deacetylation

## Abstract

Fine particulate matter (PM_2.5_) exposure is correlated with the risk of developing cardiac fibrosis. Melatonin is a major secretory product of the pineal gland that has been reported to prevent fibrosis. However, whether melatonin affects the adverse health effects of PM_2.5_ exposure has not been investigated. Thus, this study was aimed to investigate the protective effect of melatonin against PM_2.5_‐accelerated cardiac fibrosis. The echocardiography revealed that PM_2.5_ had impaired both systolic and diastolic cardiac function in ApoE^−/−^ mice. Histopathological analysis demonstrated that PM_2.5_ induced cardiomyocyte hypertrophy and fibrosis, particularly perivascular fibrosis, while the melatonin administration was effective in alleviating PM_2.5_‐induced cardiac dysfunction and fibrosis in mice. Results of electron microscopy and confocal scanning laser microscope confirmed that melatonin had restorative effects against impaired mitochondrial ultrastructure and augmented mitochondrial ROS generation in PM_2.5_‐treated group. Further investigation revealed melatonin administration could significantly reverse the PM_2.5_‐induced phenotypic modulation of cardiac fibroblasts into myofibroblasts. For the first time, our study found that melatonin effectively alleviates PM_2.5_‐induced cardiac dysfunction and fibrosis via inhibiting mitochondrial oxidative injury and regulating SIRT3‐mediated SOD2 deacetylation. Our findings indicate that melatonin could be a therapy medicine for prevention and treatment of air pollution‐associated cardiac diseases.

## 1 INTRODUCTION

Currently, air pollution is a major environmental risk to human health. Air particulate matter (PM) is considered as the main atmospheric pollutant which cause about 7 million deaths per year in the world.^1^ In contrary, the mortality of various diseases, including stroke, heart disease, lung cancer, and asthma, could be declined significantly by reducing air pollution levels.^2^, ^3^ In China, although the air pollution has been controlled effectively by central government and the level of PM_2.5_ has been decreased year by year, the PM_2.5_‐associated premature deaths are still elevated gradually.^4^ Among them, the mortality of cardiovascular disease is up to 60% approximately in all diseases that associated with PM_2.5_. Reports from the American Heart Association (AHA) confirm that every 10 μg/m^3^ elevation in PM_2.5_ concentration lead to 8%‐18% increase in cardiovascular mortality.^5^

Extensive epidemiological evidences reveal that PM_2.5_ is linked to hypertension, thrombosis, atherosclerosis, stroke, and heart failure.^6^ However, few studies focus on the cardiac fibrosis triggered by PM_2.5_, and the underlying mechanism is largely unknown. Cardiac fibrosis is defined as the excessive accumulation of cardiac fibroblasts accompanied by deposition of extracellular matrix and contributes to both systolic and diastolic dysfunction in many cardiac pathophysiologic conditions.^7^, ^8^ It could be further subdivided into reactive fibrosis and reparative fibrosis.^9^ Because of the negligible regenerative capacity of adult mammalian heart, death of massive cardiomyocytes results in reparative fibrosis. On the other hand, other chronic stimulatory factors elicit reactive fibrosis that includes perivascular and interstitial fibrosis.^9^ Epidemiologic studies have already supported the association between adverse cardiac remodeling and ambient air pollution.^10^ Consistently, several studies that show PM_2.5_ exposure induced cardiac phenotypic changes involved cardiac fibrosis, cardiac hypertrophy, cardiomyocyte apoptosis, inflammatory reaction, etc.^11^, ^12^ Notwithstanding, there have been only limited data suggest the underlying mechanism of PM_2.5_‐induced cardiac injury.

Mitochondria are vital organelles involved in maintaining cellular homeostasis. Accumulating evidence has reported that the mitochondrion could be a sensitive target of both environmental stress factors and oxidative stress, one of them represented by PM_2.5_.^13^, ^14^, ^15^, ^16^ Mitochondria are essential organelles to maintain the fundamental physiology in heart tissues through energy metabolism and catabolic oxidative reactions.^17^, ^18^ The processes of mitochondrial metabolism are dependent on the two NAD+‐dependent enzyme families, sirtuins (SIRTs) and poly (ADP‐ribose) polymerases (PARPs).^19^, ^20^ The SIRTs family (SIRT1‐SIRT7) could affect their target proteins activities by deacetylation which transfers an acetyl group from their target proteins to ADP‐ribose moiety of NAD+ to form 2‐O‐acetyl‐ADP‐ribose and NAM.^21^, ^22^ SIRT3 primarily localizes to the mitochondria which plays a critical role in regulating mitochondrial oxidative stress and has been demonstrated involved in multiple cardiovascular diseases.^23^ Melatonin is a well‐known powerful potent antioxidants and free radical scavenger.^24^, ^25^ Melatonin is able to reverse the lung injury enhanced or induced by PM_2.5_ exposure by attenuating oxidative stress.^26^, ^27^ However, the ability of melatonin to reverse or prevent PM_2.5_‐induced cardiac fibrosis remains unknown.

Hyperlipidemia is a risk factor for diverse cardiovascular diseases and a focus of primary prevention research.^28^ Epidemiological studies have shown that participants with hyperlipidemia exposure to PM_2.5_ may make them more susceptible to a higher risk of developing CVDs.^29^, ^30^, ^31^ Therefore, we need a more thorough understanding of the potential mechanisms in order to develop preventive intervention strategies. Consequently, we use an ApoE^−/−^ mouse model which is a mature animal model of hyperlipidemia. In the present study, we investigated whether melatonin could ameliorate PM_2.5_‐induced cardiac fibrosis and what mechanism involved in this progress. The data presented in this study indicate that either in vivo or in vitro supplementation of melatonin significantly reduced the mitochondrial ROS generation and cardiac fibrosis. Furthermore, we revealed that the protective action of melatonin was closely associated with SIRT3‐mediated SOD2 deacetylation. These data provide a new underlying molecular mechanism of melatonin that may be utilized for future both the prevention and treatment of PM_2.5_‐induced cardiotoxicity.

## 2 MATERIALS AND METHODS

### 2.1 Collection and preparation of PM_2.5_ samples

The PM_2.5_ samples were collected for an entire year from January to December in 2017 with a large‐capacity air particle sampler (TH‐1000C). The size distribution, morphology, and component of the constituents of PM_2.5_ have been characterized in our previous study.^32^, ^33^

### 2.2 Experimental animals and treatments

The animal experiments were approved by Animal Care and Use Committee of Capital Medical University (ethics number: AEEI‐2016‐076) and performed in accordance with guidelines. A total of sixty male ApoE^−/−^ mice aged 7 weeks were obtained from the Vital River Laboratory Animal Technologies Co. Ltd. (Beijing, China). All mice were maintained under standard conditions (20‐24°C, 40%‐60% humidity, 12/12‐hour light/dark cycle). After 1 week of acclimation, mice were fed with a high‐fat diet containing 0.15% cholesterol and 21% fat. After 4 weeks of high‐fat diet, mice were daily orally gavage with melatonin (20 mg/kg·bw) and twice a week given intratracheal instillation of PM_2.5_ (5 mg/kg·bw) for 4 weeks. The control mice received intratracheal instillation of a corresponding volume of unexposed filters (blank filters) eluted saline. The vehicle mice were given an equal volume of sterile water by gavage.

Melatonin (Sigma) was dissolved in absolute ethanol as a fresh stock and diluted in sterile water to a final concentration of 0.5% ethanol. The lyophilized PM_2.5_ samples were exposed to ultraviolet radiation for 6 hours and then were mixed with sterilized saline and sonicated for 30 minutes to suspend. The intratracheal instillation dosage of PM_2.5_ was estimated from interim target‐1 (IT‐1) for annual mean of PM_2.5_ concentration (35 μg/m^3^), as recommended by the World Health Organization air quality guidelines. In addition to ambient air quality standard, mice respiratory physiological parameters and the 100‐fold uncertainty factor was used to determine exposure dose.

### 2.3 Experimental design

The mice were randomly divided into four groups (n = 15 per group): (a) the Con‐Veh group was given saline by intratracheal instillation and sterile water by gavage; (b) the Con‐Mel group was given saline by intratracheal instillation and melatonin by gavage; (c) the PM_2.5_‐Veh group received intratracheal instillation of PM_2.5_ and gavage with sterile water; and (d) the PM_2.5_‐Mel group was administered with PM_2.5_ by intratracheal instillation and melatonin by gavage.

### 2.4 Echocardiographic assessment

As previously described, the mice were anesthetized with tribromoethanol (350 mg/kg, i.p.) and echocardiographic images were obtained using the Vevo2100 imaging system (FUJIFILM Visual Sonics).^33^ M‐mode was used to determine systolic and diastolic left ventricle wall thickness, internal dimensions, left ventricle volume, and left ventricle mass. The fractional shortening (FS) and ejection fraction (EF) were calculated to reflect systolic function. Left ventricular diastolic dysfunction was assessed on the basis of Doppler patterns of mitral inflow. The parameters were analyzed including ejection time (ET), E/A ratio, isovolumic contraction time (IVCT), and isovolumic relaxation time (IVRT).

### 2.5 Histopathological analysis

Mouse hearts were isolated and washed with prechilled phosphate‐buffered saline (PBS) three times, followed by paraformaldehyde fixation (4%) and paraffin embedding. The paraffin‐embedded sections (5 μm thick) were stained with standard Masson's trichrome and α‐SMA (Abcam) to determine the degree of cardiac fibrosis. The paraffin sections were also stained with Texas Red‐X conjugated Wheat Germ Agglutinin (WGA; Invitrogen) to analyze the cross‐sectional area of individual cardiomyocytes.

### 2.6 Ultrastructural observation by TEM

The ultrastructural observation of myocardium was performed using a transmission electron microscopy accordingly to routine procedure. In brief, mouse hearts were perfused with precooled PBS and then immediately fixed in 2.5% glutaraldehyde. After 6 hours fixation, the process of washing, dehydration, embedding, ultrathin sections were prepared for analysis in a transmission electron microscope (TEM; JEOL JEM2100).

### 2.7 Evaluation of mitochondrial ROS generation in heart

To evaluate mitochondrial reactive oxygen species (ROS) levels, the heart tissues were prepared into frozen sections for MitoSOX staining (Invitrogen). Fluorescence images were captured using a Leica laser scanning confocal microscope (LSCM, TCS SP8 STED; Leica), and images were processed using Image‐Pro Plus 6.0 Software.

### 2.8 Assessment of oxidative stress in heart

The 3′‐nitrotyrosine (3′‐NT) and 4‐hydroxynonenal (4‐HNE) content in heart tissues were determined using ELISA kits (mlbio), respectively, according to the manufacturer's protocol. The levels of total glutathione/oxidized glutathione (GSH/GSSG) in heart tissues were determined by a glutathione Assay kit (Nanjing Jiancheng Bioengineering Institute) as commercially recommended by the manufacturer's protocols.

### 2.9 Cell culture and PM_2.5_ exposure

The human embryonic heart fibroblast cell line was obtained from the Kunming Cell Bank of Chinese Academy of Sciences. The cardiac fibroblasts were grown in DMEM/F12 (Corning) supplemented with 10% fetal bovine serum (Corning) and 1% penicillin and streptomycin (Key Gen). The cardiac fibroblasts were incubated at 37°C in 5% CO_2_ in a humidified environment. The cardiac fibroblasts were seeded on 6 well culture plates and grown to 70%‐80% density over 24 hours. Myofibroblast differentiation was induced using 10 µmol/L Angiotensin II (Sigma). In all experiments, FBS was reduced to 1% 12 hours during treatment with AngII. After cells were differentiated for 12 hours, cells were exposed to PM_2.5_ and/or melatonin for another 24 hours.

### 2.10 Assessment of cell viability

The cardiac fibroblasts were exposed to various concentrations of PM_2.5_ (0, 12.5, 25, 50, and 100 μg/mL) and melatonin (0, 25, 50, 75, 100, 125, and 150 μmol/L). Cell viability was measured after 24 hours exposure by cell counting assay (CCK‐8, Tongren, Japan) according to the manufacturer's instructions.

### 2.11 Measurement of mitochondrial ROS generation

Mitochondria‐associated ROS production in the cardiac fibroblasts was measured by MitoSOX (Invitrogen) staining using a flow cytometric analysis. The cells after treatment were incubated with 5 μmol/L MitoSOX for 30 minutes at 37°C in dark. After staining, cells were washed twice with warm PBS and at least 1 × 10^4^ cells were analyzed using a NovoCyte flow cytometer (ACEA Biosciences).

### 2.12 Immunofluorescence staining

The identification of the cardiac fibroblasts was performed using immunofluorescence staining with anti‐vimentin antibody. To evaluate the phenotypic change of cardiac fibroblast to the myofibroblast phenotype after treatment of PM_2.5_, immunofluorescence was used to monitor increased expression of α‐smooth muscle actin (α‐SMA). Cells were placed on glass slides in 12‐well plates and cultured at 5% CO_2_, 37°C. The cells reached 70%‐80% density and were treated with PM_2.5_ for 24 hours and then washed twice with precooling PBS and fixed with 4% paraformaldehyde for 10 minutes at room temperature, followed by permeabilized with 0.1% Triton X‐100. Immunofluorescence staining was performed by incubating the cardiac fibroblasts with vimentin and α‐SMA (Abcam) primary antibodies overnight at 4°C, following treated with fluorescently conjugated secondary antibodies (Abcam) for 60 minutes at room temperature. The cardiac fibroblasts were incubated with mounting medium contained DAPI (Vector) to highlight the cellular nucleus. Finally, the stained cells were visualized and photographed using LSCM (TCS SP8 STED; Leica).

### 2.13 SIRT3 deacetylation activity measurement

Cells and tissues mitochondrial fractionations were purified by using the mitochondrion isolation Kit (Key Gen) with the manufacturer's instructions. The homogenization was performed using a glass tissue homogenizer within mitochondrial isolation buffer. The fresh pellets were separated by centrifugation and used for testing. The deacetylase activity of SIRT3 was measured by a SIRT3 Fluorimetric Activity Assay Kit (BPS bioscience) following the manufacturer's protocol. Fluorescent intensity was measured using a SpectraMax Multi‐Mode Microplate Reader (Molecular Devices) at excitation wavelength of 350 nm.

### 2.14 Measurement of SOD2 enzyme activity

Mitochondrial superoxide dismutase 2 (SOD2) activity was assayed using a SOD1/SOD2 Assay Kit (Beyotime Company) following the manufacturer's instructions. The specific activity of SOD2 was expressed as U/mg of total protein.

### 2.15 qRT‐PCR assay

Total RNA was extracted from the cardiac tissue using the TRIzol reagent (Invitrogen) according to the instructions provided by the manufacturer. The isolated RNA was reverse transcribed into cDNA by the Primescript RT Reagent Kit (TaKaRa Bio, China). Quantitative real‐time polymerase chain reaction (qRT‐PCR) was conducted using SYBR Green Mix kit (TaKaRa Bio) on an Eppendorf machine (Eppendorf Mastercycler ep realplex). GAPDH was used as an internal reference, and the relative expressions of genes were calculated using the 2^−∆∆Ct^ method. All primer sequences were listed in Table 1.

**TABLE 1:** Primers used for real‐time PCR

Primer	Forward primer (5′‐3′)	Reverse primer (5′‐3′)
Col I	TGGTCCTGCTGGTCCTGCTG	CTGTCACCTTGTTCGCCTGTCTC
Col III	TCTCCTGGTGCTGCTGGTCAC	TCCATGTGGTCCAACTGGTCCTC
ANP	AAGAACCTGCTAGACCACCTGGAG	TGCTTCCTCAGTCTGCTCACTCAG
BNP	GGAAGTCCTAGCCAGTCTCCAGAG	GCCTTGGTCCTTCAAGAGCTGTC
α‐MHC	CAGAACACCAGCCTCATCAACCAG	TTCTCCTCTGCGTTCCTACACTCC
β‐MHC	GCAAGACGGTGACTGTGAAGGAG	GGTTGACGGTGACGCAGAAGAG
AERCA2a	CTGCTGTCATCACCACCTGCTTAG	TCCAGAATGAACATCCTGCACACG
GAPDH	GGTTGTCTCCTGCGACTTCA	TGGTCCAGGGTTTCTTACTCC

### 2.16 Western blot analysis

Total protein samples were extracted from heart tissues and the cardiac fibroblasts by a protein extraction kit (Key Gen). The concentration of proteins was quantified using a BCA protein assay kit (Dingguo Biotechnology). We applied 30 µg of protein samples to a 10%‐12% sodium dodecyl sulfate polyacrylamide gel electrophoresis (SDS‐PAGE) and transferred to the nitrocellulose membranes (Pall Corp.). The membranes were incubated with primary and then fluorescently conjugated secondary antibodies. The protein expressions were detected using the Li‐Cor Odyssey system (Li‐Cor Biosciences) and quantified using Image Studio Software (Li‐Cor Biosciences).

### 2.17 Statistical analysis

All data were analyzed using SPSS 24.0 software, and continuous variables were expressed as the means ± SD. Statistical analysis was performed with Student's *t* test or one‐way ANOVA with Bonferroni's post hoc analysis (normality and equal variance passed). Kruskal‐Wallis test was applied for nonparametric (normality and/or equal variance failed) data. A *P* value < .05 was considered statistically significance.

## 3 RESULTS

### 3.1 Melatonin alleviated cardiac dysfunction caused by PM_2.5_ in ApoE^−/−^ mice

**FIGURE 1:**
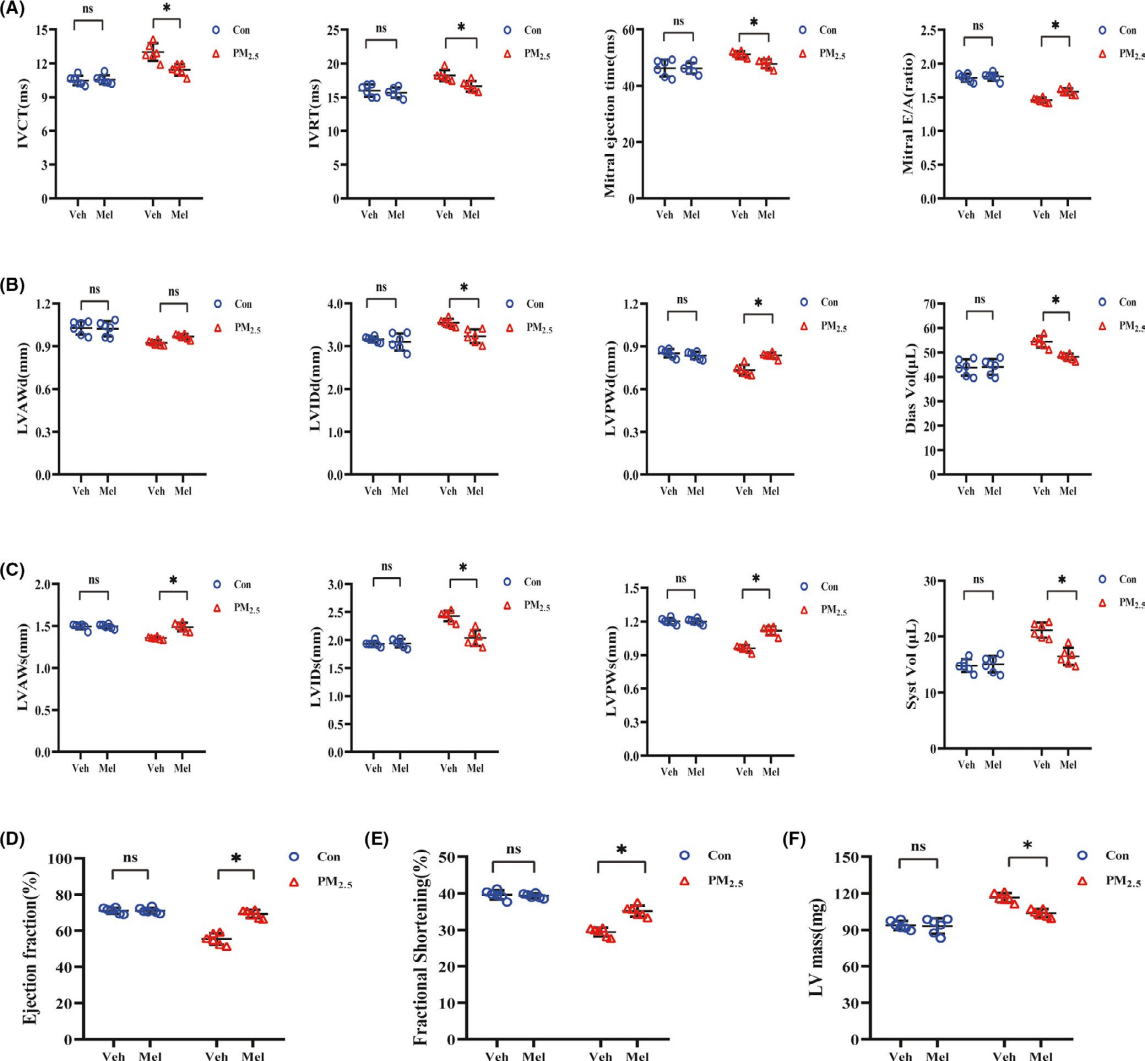
Melatonin treatment attenuated cardiac systolic and diastolic dysfunction after exposure to PM_2.5_. A, Left ventricular diastolic function after melatonin treatment. B, Diastolic left ventricular wall thickness and volume after melatonin treatment. C, Systolic left ventricular wall thickness and volume after melatonin treatment. D, Ejection fraction with melatonin treatment. E, Fractional shortening with melatonin treatment. F, Left ventricular mass with melatonin treatment. Data are expressed as the means ± SD; n = 6 in each group. *P < .05

Results of diastolic and systolic dysfunction from transthoracic echocardiographic analysis were shown in Figure 1. We evaluated left ventricle diastolic function by mitral valve Doppler echocardiography (Figure 1A). There was no significant difference identified between the Con‐Veh group and Con‐Mel group. However, when analysis was performed within PM_2.5_ treatment group, some differences were observed. After exposure to PM_2.5_, the ApoE^−/−^ mice had higher IVCT values, higher IVRT values, prolonged mitral ejection time, and lower E/A ratio. However, these alterations were significantly improved after the treatment of melatonin. Exposure to PM_2.5_ had been linked to a reduction in anterior and posterior wall thickness along with significant increase in internal diameter and ventricular volume (both systolic and diastolic), which ultimately lead to the deterioration of cardiac function (Figure 1B,C). It was unexpected that melatonin treatment markedly improved these indexes without altering diastolic anterior wall thickness. Left ventricle FS and EF were used as indices of the systolic function of the heart which represented hallmarks of compromised state of cardiac function. Echocardiography also showed a remarkable decrease of EF and FS in the PM_2.5_‐exposed mice (Figure 1D). Additionally, both EF and FS were increased in PM_2.5_‐treated mice with melatonin administration compared to untreated PM_2.5_‐exposed mice. The increase in left ventricular mass is a critical indicator of left ventricular hypertrophy or left ventricular remodeling. Measurement of the left ventricular mass revealed that left ventricle mass was increased in response to PM_2.5_, and melatonin treatment significantly decreased left ventricular weight compared with that following PM_2.5_ treatment (Figure 1D). Taken together, these results indicated that PM_2.5_ exposure could induce cardiac dysfunction while melatonin treatment provided beneficial effects on heart.

### 3.2 Melatonin exerted an opposing function in PM_2.5_‐induced cardiac hypertrophy and fibrosis

**FIGURE 2:**
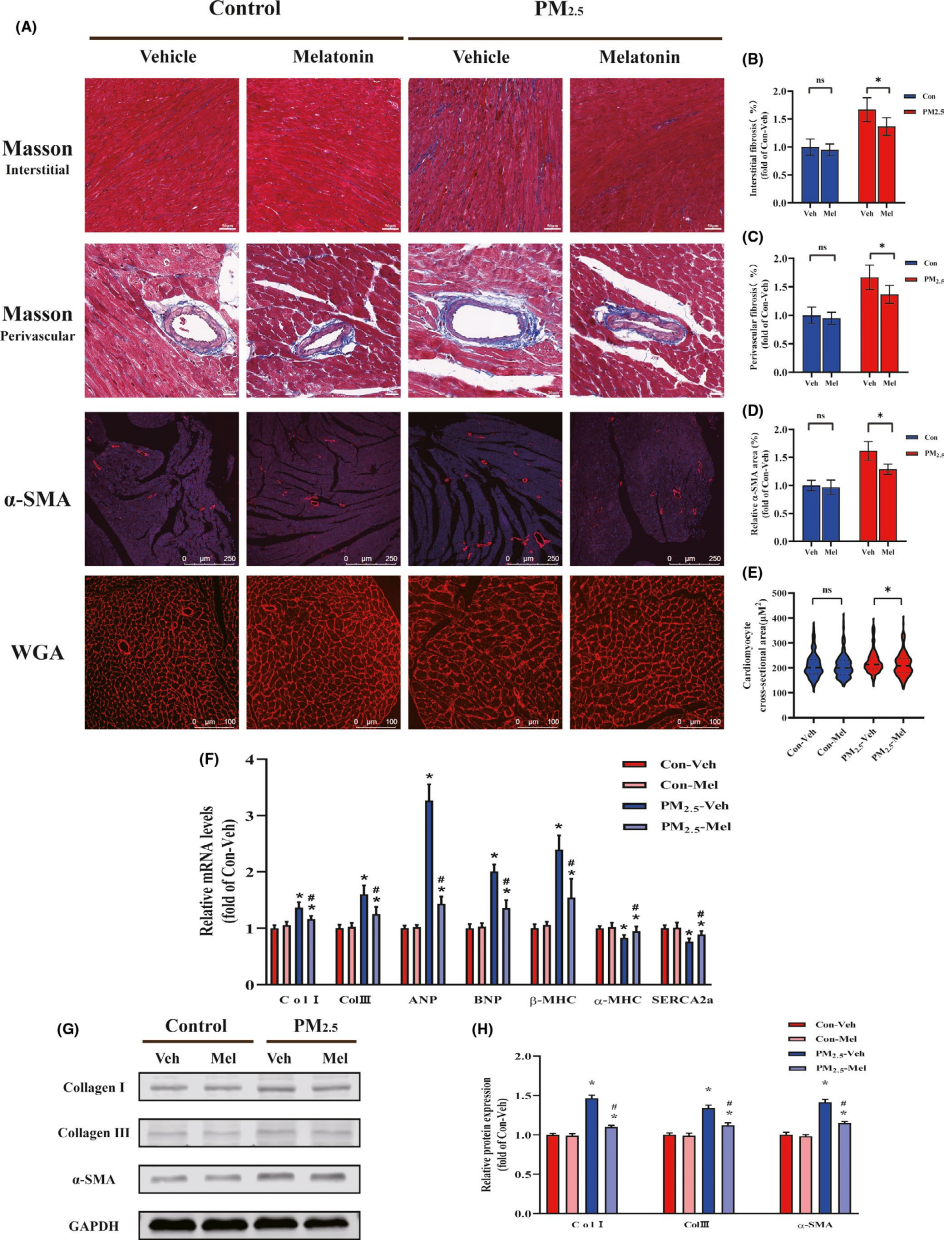
Melatonin ameliorated PM_2.5_‐induced myocardial hypertrophy and fibrosis. A, Representative images of histopathological changes in cardiac tissues. Masson's trichrome staining in myocardial interstitial (scale bar: 50 μm); Masson's trichrome staining in myocardial perivascular (scale bar: 20 μm); Immunohistochemically staining of α‐SMA in heart sections (scale bar: 250 μm); Wheat germ agglutinin staining in heart sections (scale bar: 100 μm). B, Quantitative analysis of myocardial interstitial fibrosis. C, Quantitative analysis of myocardial perivascular fibrosis. D, Quantitative analysis of α‐SMA‐positive area percentages of hearts. E, Quantitative analysis of cardiomyocyte cross‐sectional area. F, The mRNA expression after melatonin treatment. G, Representative Western blot pictures. H, The quantitative analysis of protein levels. Data are expressed as the means ± SD; n = 6 in each group. *P < .05 compared to Con‐Veh group;^#^ *P* < .05 compared to PM_2.5_‐Veh group

It has been shown that PM_2.5_ exposure deteriorated overt cardiac fibrosis. In the present study, representative Masson trichrome‐stained histological sections showing PM_2.5_‐exposured markedly exacerbated the development of myocardial interstitial fibrosis and perivascular fibrosis (Figure 2A,C). Following melatonin treatment, both interstitial and perivascular fibrotic areas were decreased in heart. α‐SMA is a key marker of cardiac fibrosis. The results showed that daily gavage administration of melatonin significantly attenuated the increased α‐SMA‐positive area in the heart tissue of PM_2.5_‐exposed groups (Figure 2A,D). Unexpectedly, compared with control group, exposure to PM_2.5_ group displayed an obvious cardiac hypertrophy by WGA staining (Figure 2E). Strikingly, melatonin administration attenuated cardiomyocyte hypertrophy in PM_2.5_‐exposed mice. Subsequent analysis of the mRNA expression levels of hypertrophy‐related and fibrosis‐related markers in mouse hearts was measured after PM_2.5_ exposure (Figure 2F). PM_2.5_ exposure resulted in significant increases the expression of cardiac hypertrophy markers (ANP, BNP, β‐MHC, α‐MHC, SERCA‐2a) and myocardial fibrosis indicators (Collagen I and Collagen III), whereas melatonin treatment reversed these effects. Cardiac fibrosis plays an important role in cardiac functional changes. In order to further validate the findings, fibrosis‐associated protein expression levels were determined by Western blotting. PM_2.5_ administration resulted in an increase in the accumulations of collagen and α‐SMA, and a subsequent analysis showed that melatonin inhibited collagen I, collagen III, and α‐SMA expression (Figure 2G,H). The results above show that PM_2.5_‐induced cardiac hypertrophy and fibrosis, melatonin treatment had a better alleviating effect on heart injury.

### 3.3 Melatonin reduced mitochondrial oxidative stress after PM_2.5_ treatment

**FIGURE 3:**
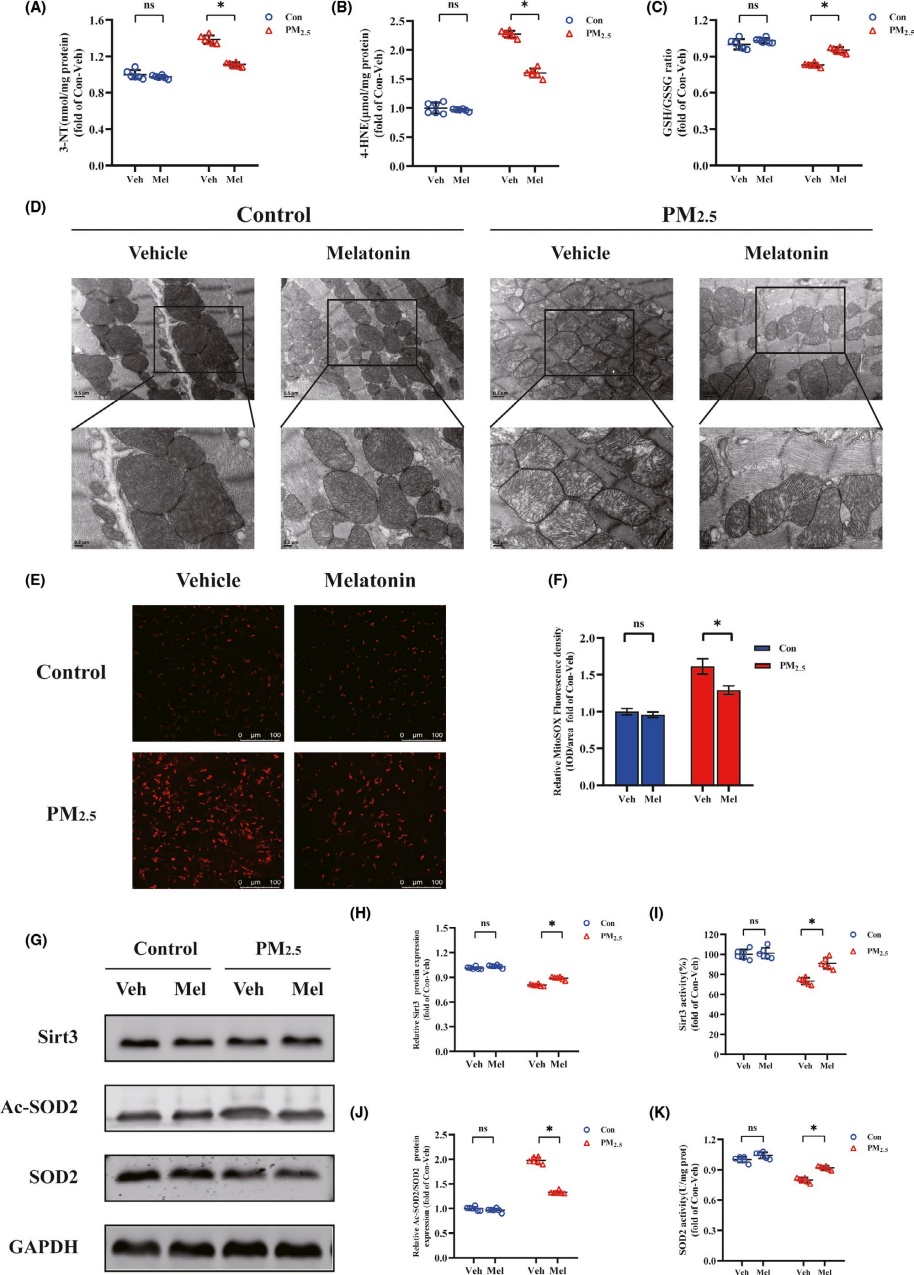
Melatonin alleviated PM_2.5_‐induced cardiac mitochondrial oxidative damage. A, The level of 3′‐NT. B, The level of 4‐HNE. C, The level of GSH/GSSG. D, Representative pictures of myocardial tissue by transmission electron microscope. The blow panels are high magnification (scale bar: 0.2 μm) images corresponding to the upper panels (scale bar: 0.5 μm). E, MitoSOX staining of heart tissues (scale bar: 100 μm). F, Mean fluorescence intensities of heart. G, Representative Western blot pictures. H, The quantitative analysis of SIRT3 protein levels. I, The activity of SIRT3. J, The quantitative analysis of SOD2 acetylation. K, The activity of SOD2. Data are expressed as the means ± SD; n = 6 in each group. *P < .05

Multiple studies have suggested that PM_2.5_ contributes to cardiac hypertrophy and fibrosis through mechanisms that increase oxidative stress. To determine cardiac oxidative stress after PM_2.5_ exposure, the accumulation of oxidative and nitrosative damage products including protein‐nitration‐related 3′‐NT and lipid peroxidation‐related 4‐HNE was assessed (Figure 3A,B). According to the quantitative analysis, the expression levels in heart of mice following PM_2.5_ treatment were up to 1.5 times higher for 3NT and two times higher for 4HNE. Meanwhile, the extent of oxidative stress was also assessed by the glutathione redox ratio (GSH/GSSG; Figure 3C). As expected, the exposure to PM_2.5_ reduced the ratio of GSH/GSSG in heart compared to that control. Unexpectedly, these indicators of oxidative damage alterations were reversed after melatonin supplementation. Mitochondria play a key role in the development of oxidative stress. Meanwhile, myocardial tissue is rich in mitochondria and relies strongly on mitochondrial function. To assess the effects of PM_2.5_ on myocardial mitochondria, TEM was used to observe the ultrastructure of mitochondria (Figure 3D). TEM images showed myocardial mitochondria from PM_2.5_‐exposed group had suffered substantial structural damage, with the fragmented mitochondrial membranes; the mitochondria were significantly swollen, and crista space was widened and broken; the mitochondria were disordered and vacuolated. These results could be greatly improved by melatonin treatment. To investigate the effects of PM_2.5_/melatonin on myocardial mitochondrial ROS generation, the heart sections were stained with MitoSOX to evaluate the mitochondrial ROS levels. PM_2.5_ treatment could trigger the mitochondrial ROS generation which could further relief by melatonin treatment (Figure 3E,F). In summary, these findings indicated that melatonin attenuated PM_2.5_‐induced myocardial oxidative stress.

### 3.4 Melatonin exerted cardioprotection via promoting SIRT3‐mediated SOD deacetylation

The deacetylase SIRT3 primarily located in mitochondria, which is strongly associated with oxidative stress and has been implicated in many cardiac‐related diseases. First of all, the protein expression of SIRT3 was measured with Western blots. The results were displayed SIRT3 protein expression was significantly decreased in PM_2.5_‐exposed group when compared to control group (Figure 3G,H). At the same time, melatonin supplementation markedly increased SIRT3 expression in PM_2.5_‐exposed group. Aside from this, the activity of SIRT3 was significantly reduced by PM_2.5_ treatment, but was profoundly potentiated in heart after melatonin treatment (Figure 3I). Mitochondrial superoxide dismutase (SOD2) is the most commonly known scavengers of ROS and an important downstream protein of the SIRT3 signaling pathway. The capacity of ROS scavenging by SOD2 is tightly regulated by enzyme activity and the degree of acetylation. The results of the current study suggested that PM_2.5_ exposure decreased SOD2 expression and increased acetylation of SOD2, whereas Ac‐SOD2/SOD2 ratio was elevated (Figure 3J). Nevertheless, melatonin significantly attenuated PM_2.5_‐induced upregulation of acetylated‐SOD2 and restored the SOD2 protein levels. In addition, PM_2.5_ treatment significantly reduced the activity of antioxidant enzyme SOD2, while melatonin significantly increased the SOD2 activity (Figure 3K). All the above results demonstrated that melatonin supplementation conferred cardioprotection against heart injury triggered by PM_2.5_.

### 3.5 PM_2.5_ induced mitochondrial ROS production in cardiac fibroblasts

**FIGURE 4:**
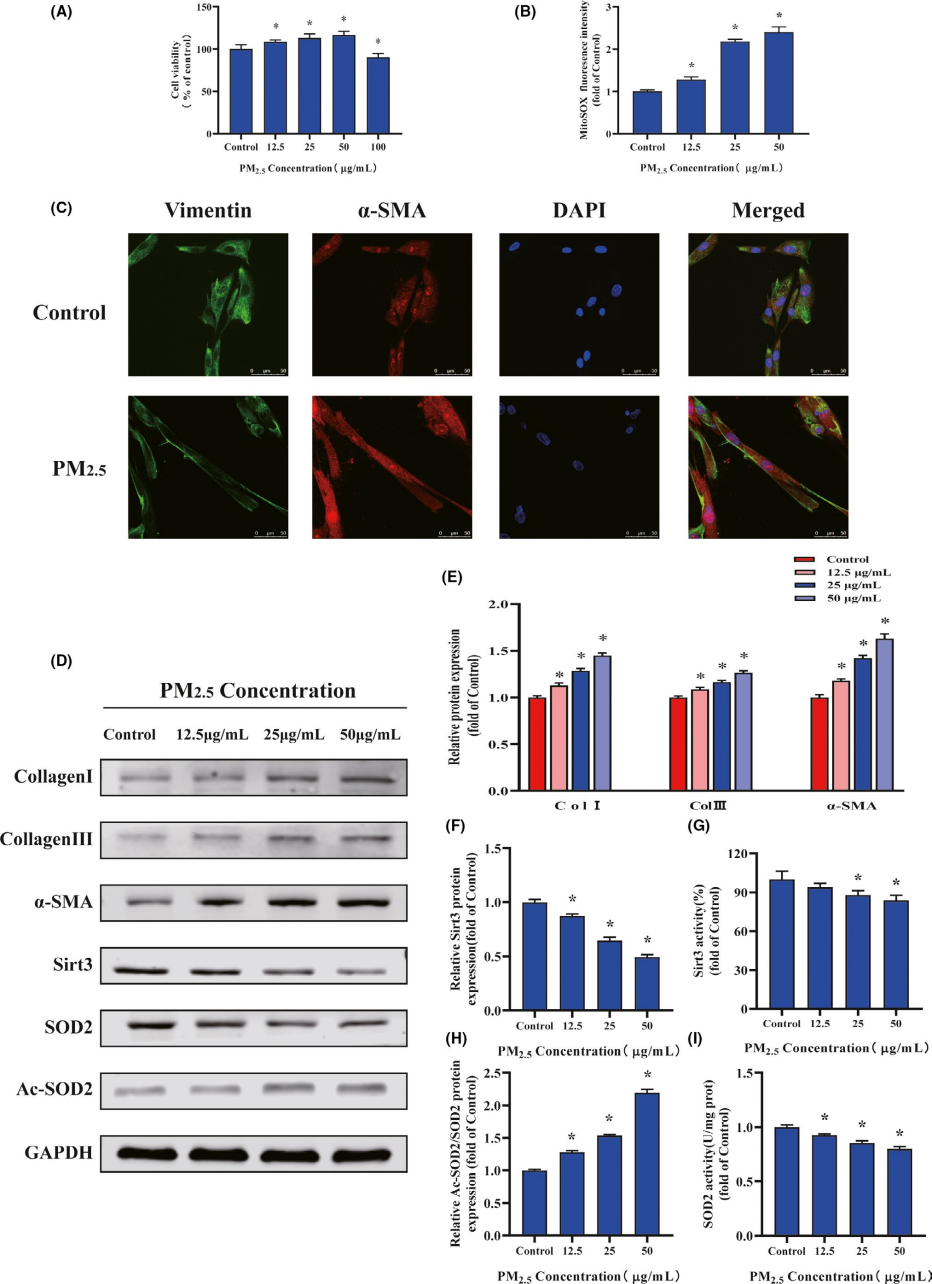
PM_2.5_regulated cardiac myofibroblast conversion via increased mitochondrial reactive oxygen levels and SOD2 acetylation, decreased SIRT3 expression and activity. A, Cardiac fibroblast cell viability after PM_2.5_ **‐**treated. B, Quantification analysis of fluorescence intensity obtained from flow cytometry. C, Representative images of cardiac fibroblast α‐SMA and Vimentin immunostaining. D, Representative Western blot pictures. E, The quantitative analysis of collagen‐I, collagen‐III, and α‐SMA protein levels. F, The quantitative analysis of SIRT3 protein levels. G, The activity of SIRT3. H, The quantitative analysis of SOD2 acetylation. I, The activity of SOD2. Data are expressed as the means ± SD from three independent experiments. *P < 0.05 compared to control group

The effect of PM_2.5_ on cell viability in human cardiac myofibroblasts was assessed by CCK‐8 assay. As presented in Figure 4A, the cell viability of the cardiac fibroblasts first elevated and then declined with the increasing concentration of PM_2.5_ treatment. Treatment with between 12.5 and 50 µg/mL PM_2.5_ for 24 hours increased the cell viability (>100%), when compared with untreated cells. Meanwhile, the concentration of PM_2.5_ (25 μg/mL) was selected for subsequent experiments. Mitochondrial ROS caused by PM_2.5_ was analyzed by incubation with MitoSOX for 30 minutes followed by quantification of flow cytometry analysis (Figure 4B). Compared to the control group, the mitochondrial ROS fluorescence intensity was significantly increased in PM_2.5_ treated cardiac fibroblasts, which was demonstrated in a dose‐dependent fashion with the PM_2.5_ concentration. These findings suggested that PM_2.5_ treatment lead to elevated mitochondrial ROS production in cardiac fibroblasts which presented cytotoxicity.

### 3.6 PM2.5 modulated phenotype transformation of fibroblasts via oxidative stress‐regulated mitochondrial SIRT3 signaling pathway

**FIGURE 5:**
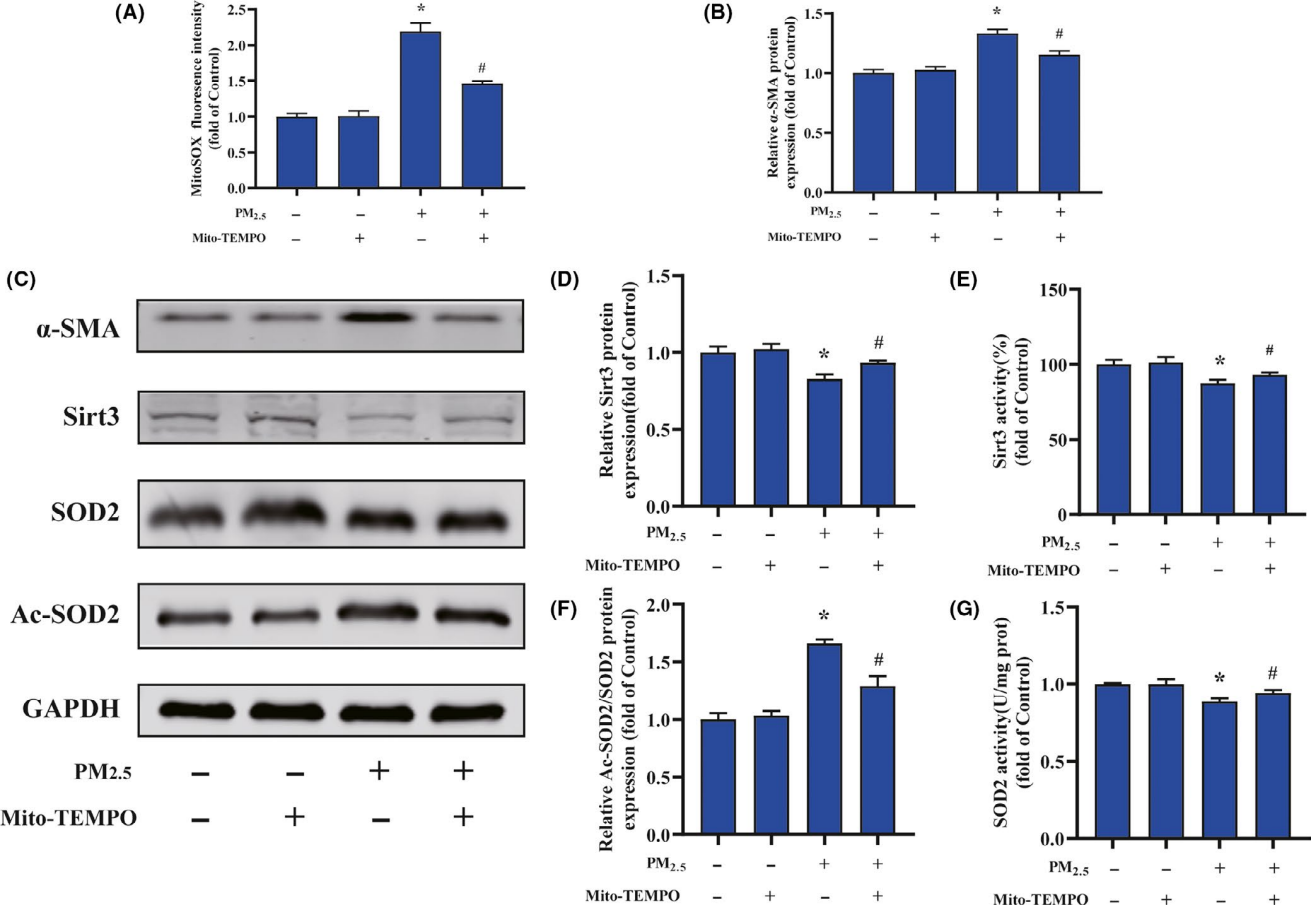
Mitochondrial‐derived ROS mediated PM_2.5_‐induced cardiac myofibroblast conversion. A, Quantification analysis of fluorescence intensity obtained from flow cytometry. B, The quantitative analysis of α‐SMA protein levels. C, Representative Western blot pictures. D, The quantitative analysis of SIRT3 protein levels. E, The activity of SIRT3. F, The quantitative analysis of SOD2 acetylation. G, The activity of SOD2. Data are expressed as the means ± SD from three independent experiments. *P < .05 compared to control group;^#^ *P* < .05 compared to PM_2.5_‐treated group

To determine whether the PM_2.5_ affected fibroblast to myofibroblast transition, immunofluorescence staining was performed (Figure 4C). Representative images demonstrated that the cardiac fibroblasts were positive staining for vimentin and the expression of α‐SMA was significantly activated in PM_2.5_ treatment group. In agreement with the immunofluorescence data, Western blot analysis demonstrated that treatment with PM_2.5_ upregulated the expression of the myofibroblast markers α‐SMA in a dose‐dependent manner (Figure 4D,E). To evaluate the effect of PM_2.5_ on collagen synthesis, human cardiac fibroblasts were treated with various concentrations of PM_2.5_ (0‐50 µg/mL) for 24 hours. The quantitative measurement of the protein expression showed that type I and III collagen gradually increased as PM_2.5_ concentration progressed (Figure 4E). The relative protein expression of collagen in response to the highest concentration of PM_2.5_ treatment was approximately 1.3‐1.5 times that of control. Then, the activation of SIRT3 pathway was examined after PM_2.5_ exposure. As shown in Figure 4F,G, both expression and deacetylation activity of SIRT3 in human cardiac fibroblasts were significantly reduced in the PM_2.5_ groups compared with control groups in a dosage dependent manner. In contrast with the decreased activity of SIRT3, the fibroblasts after PM_2.5_ treatment presented increased levels of acetylated SOD2 and repressed SOD2 activity (Figure 4H,I). To obtain additional details on the fibrogenic role of mitochondrial ROS in PM_2.5_‐induced cardiac fibrosis, Mito‐TEMPO (mitochondria‐targeted antioxidant) was added to cardiac fibroblasts exposed to PM_2.5_ (Figure 5). Data analysis of flow cytometry demonstrated that the fluorescent intensity of mitochondrial ROS in Mito‐TEMPO‐treated cultures exposed to PM_2.5_ (25 μg/mL) was significantly lower than the PM_2.5_‐exposed cells (Figure 5A). As shown in Figure 5B, Mito‐TEMPO treatment significantly abolished PM_2.5_‐induced α‐SMA upregulation in cardiac fibroblasts. Meanwhile, Mito‐TEMPO treatment restored the protein expression and deacetylase activity of SIRT3 in PM_2.5_‐induced cardiac fibroblasts to control levels (Figure 5C‐E). Treatment with Mito‐TEMPO also markedly prevented the protein acetylation and restored the antioxidant activity of SOD2 in PM_2.5_‐exposed cardiac fibroblasts (Figure 5F,G). The results demonstrated that the effect of PM_2.5_ exposure on phenotype transformation in cardiac fibroblasts was mitochondrial ROS dependent, which involved in SIRT3 signaling.

### 3.7 Melatonin regulated mitochondrial redox homeostasis through SIRT3 signaling pathway in cardiac fibroblasts

**FIGURE 6:**
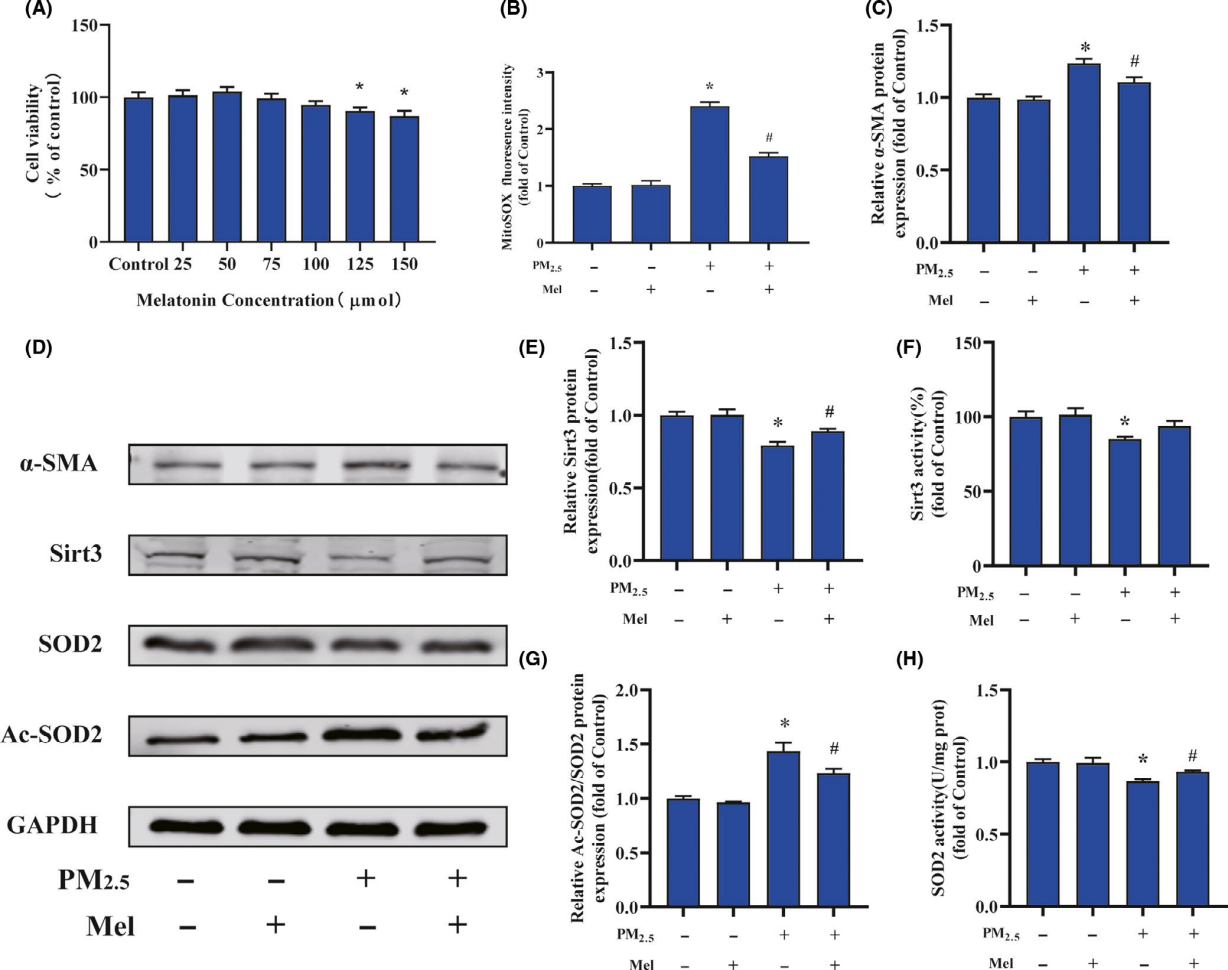
Melatonin alleviated the oxidative stress, SIRT3 impairment after PM_2.5_treatment in vitro. A, Cardiac fibroblast cell viability after melatonin**‐**treated. B, Quantification analysis of fluorescence intensity obtained from flow cytometry. C, The quantitative analysis of α‐SMA protein levels. D, Representative Western blot pictures. E, The quantitative analysis of SIRT3 protein levels. F, The activity of SIRT3. G, The quantitative analysis of SOD2 acetylation. H, The activity of SOD2. Data are expressed as the means ± SD from three independent experiments. *P < .05 compared to control group;^#^ *P* < .05 compared to PM_2.5_‐treated group

**FIGURE 7:**
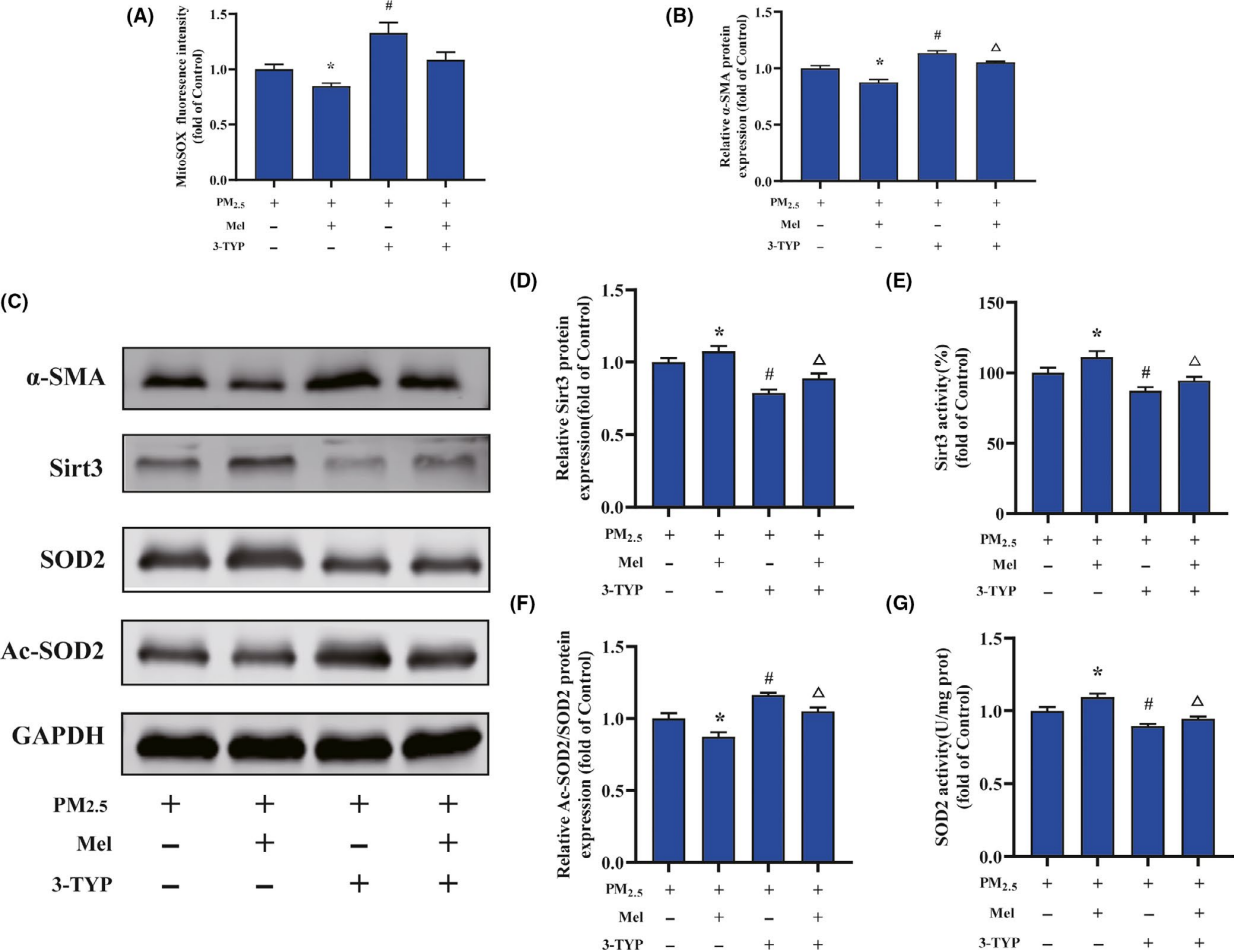
3‐TYP pretreatment abolished the melatonin‐suppressed cardiac myofibroblast conversion induced by PM_2.5_treatment. A, Quantification analysis of fluorescence intensity obtained from flow cytometry. B, The quantitative analysis of α‐SMA protein levels. C, Representative Western blot pictures. D, The quantitative analysis of SIRT3 protein levels. E, The activity of SIRT3. F, The quantitative analysis of SOD2 acetylation. G, The activity of SOD2. Data are expressed as the means ± SD from three independent experiments. *P < .05 compared to PM_2.5_‐treated group;^#^ *P* < .05 compared to melatonin pretreatment group;^∆^ *P * < .05 compared to 3‐TYP pretreatment group

**FIGURE 8:**
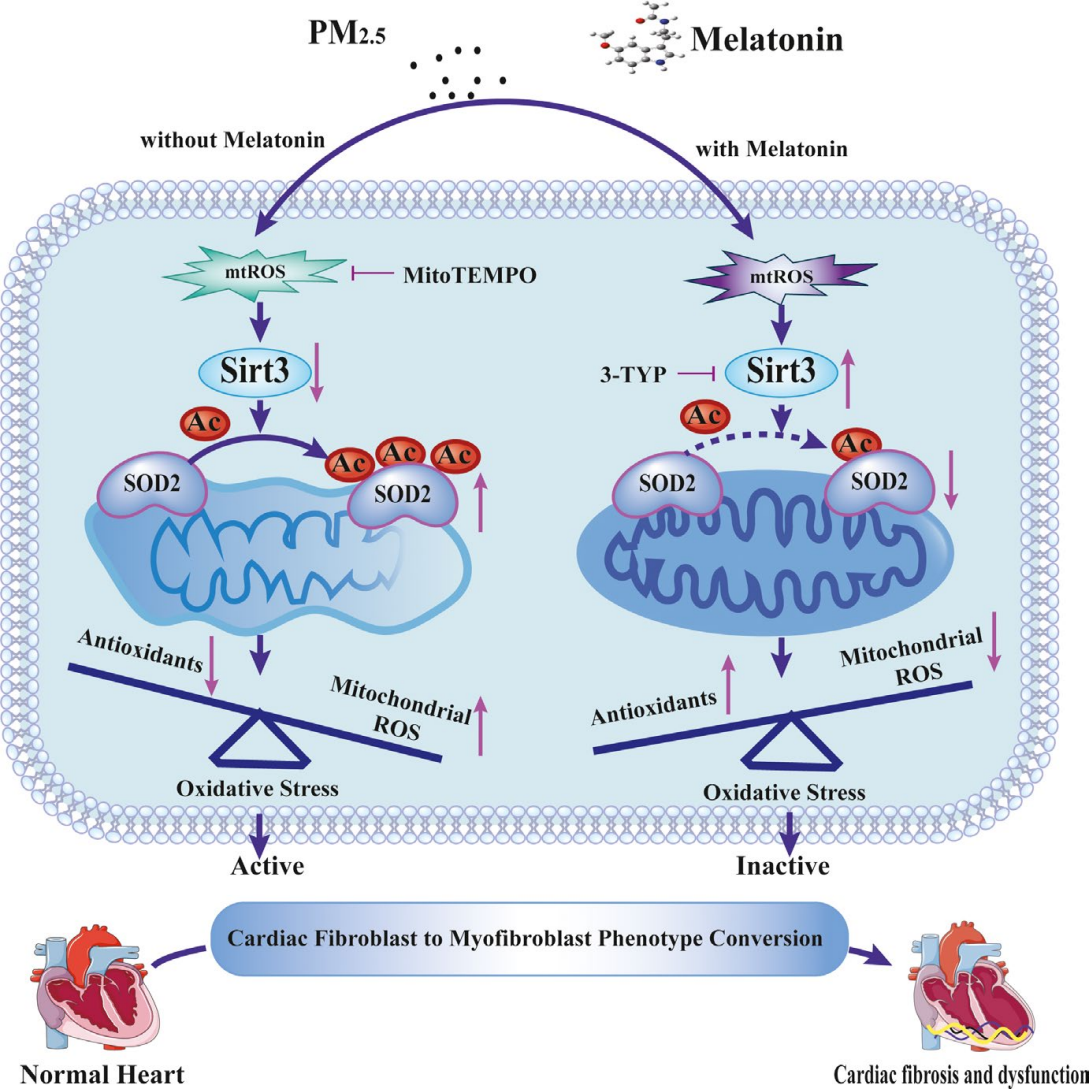
The schematic of melatonin ameliorates PM_2.5_‐induced cardiac perivascular fibrosis

To investigate the role of melatonin in regulating cardiac fibroblasts activation after PM_2.5_ treatment, we firstly evaluated the effect of melatonin on cell viability. As showed in Figure 6A, melatonin treatment dose‐dependent decreased cell viability, and the 125 and 150 μmol/mL of melatonin significantly declined the cell viability compared to the control groups. According these results, 100 μmol/L melatonin was used for further experiments. The effect of melatonin to protect against oxidative stress was investigated by measuring the levels of mitochondrial ROS production. The result indicated that melatonin treatment decreased the ROS levels of PM_2.5_‐induced cardiac fibroblasts probably by 40%, compared with PM_2.5_‐treated alone (Figure 6B). However, treatment with melatonin did not restore ROS levels to control levels. Moreover, the α‐SMA protein level indicated that melatonin attenuated the PM_2.5_‐induced fibroblast‐myofibroblast transition (Figure 6C). Similarly, the protein level of SIRT3 was altered by melatonin in the presence of PM_2.5_ (Figure 6E). Interestingly, melatonin restored the PM_2.5_‐mediated reduction in SIRT3 activity to a normal level (Figure 6F). Consistent with these results, acetylated SOD2 protein expression in PM_2.5_‐exposed cardiac fibroblasts was also markedly decreased after melatonin treatment (Figure 6G). Meanwhile, melatonin increased SOD2 activity after PM_2.5_ treatment (Figure 6H). The above results showed that melatonin reversed the transformation of cardiac fibroblasts into myofibroblast by regulating mitochondrial redox homeostasis. In order to confirm melatonin was involved PM_2.5_‐induced fibroblasts phenotype transformation through SIRT3‐mediated SOD2 deacetylation, the specific inhibitor of SIRT3 (3‐TYP) was used in this study. As shown in Figure 7A, the ROS scavenging ability of melatonin was largely abolished upon treatment with 3‐TYP in cardiac fibroblasts exposed to PM_2.5_. Besides, melatonin‐induced reduction of α‐SMA protein expression in response to PM_2.5_ was largely abolished upon treatment with 3‐TYP (Figure 7B). Simultaneously, the 3‐TYP treatment inhibited the deacetylase activity and the expression of SIRT3 compared with that in PM_2.5_‐exposed groups (Figure 7D,E). Consistent with above results, we found that inhibition of SIRT3 by 3‐TYP significantly improved the level of SOD2 acetylation and abolished melatonin protective effect in comparison with PM_2.5_ treatment group (Figure 7F). Next, the increased activities of SOD induced by melatonin in cardiac fibroblasts after PM_2.5_ exposure were also attenuated by 3‐TYP (Figure 7G). These data indicated that melatonin regulated PM_2.5_‐induced mitochondrial redox homeostasis by activating the SIRT3 signaling pathway. Taken together, the schematic of melatonin ameliorated PM_2.5_‐induced cardiac perivascular fibrosis was presented in Figure 8.

## 4 DISCUSSION

Ambient PM_2.5_ has gained considerable recognition as the greatest threat to global public health that affects human health, particularly affects the development of cardiovascular diseases.^34^ In this study, we explored that the exposure to PM_2.5_ resulted in cardiac fibrosis, especially perivascular fibrosis. In addition, melatonin had cardioprotective effects of PM_2.5_‐induced cardiac fibrosis involving the mitochondrial redox homeostasis, which reversed fibroblast to myofibroblast conversion.

In the past decade, our understanding of the pathophysiological mechanisms underlying PM_2.5_‐related effects on cardiovascular health has undergone considerable reassessment.^6^ Mechanism studies in animal models and humans have shown PM_2.5_ inhalation could result in adverse cardiac remodeling in cardiac structure and function.^35^ In this study, the echocardiography indicated that PM_2.5_ exposure induced several changes in wall thickness, ventricle diameter, and ventricular volume, which ultimately lead to the deterioration of cardiac systolic and diastolic function (Figure 1). The cardiac diastolic dysfunction phenotype is the earlier and most sensitive parameter to reflect the functional changes of left ventricle. It has been reported that mice exposed to PM_2.5_ could increase IVRT and decrease the mitral valve E/A ratio, which is consistent with our results.^36^, ^37^ Left ventricular EF and FS, as the important parameters of systolic function, have a unique role in cardiovascular disease development which have been incorporated into clinical practice guidelines.^38^, ^39^ In line with other studies, our data indicated that exposed to PM_2.5_ caused a significant decrease in both EF and FS.^12^ Left ventricular mass is an important marker for left ventricular dysfunction. In our study, an elevated left ventricular mass was observed in PM_2.5_‐exposed group (Figure 1). Here, our data found that PM_2.5_ exposure can cause markedly cardiac dysfunction accompanied by cardiac structure alterations, suggesting that pathological changes in cardiac morphology and function could be triggered by PM_2.5_ exposure.

Cardiac remodeling is the basis of heart functional and geomorphological changes involving myocyte hypertrophy and fibrosis.^40^ We further detected the cardiomyocyte cross‐sectional area and cardiac hypertrophy earlier response mRNAs, namely, ANP, BNP, α‐MHC, β‐MHC, and SERCA2a, which demonstrated that PM_2.5_ triggered cardiac hypertrophy in heart (Figure 2). On the other hand, our study revealed that PM_2.5_ treatment resulted in varied degree of severity of cardiac interstitial and perivascular fibrosis (Figure 2). According to the observed Masson staining, the cardiac fibrosis was intense in the peripheral area of the vessels whereas the interstitial fibrosis in these hearts was sporadic. Another typical marker of cardiac fibrosis is the expression of α‐SMA. We found the positive signal of α‐SMA staining was concentrated around the perivascular of heart which was consistent with the Masson staining. It had been previously reported that the early presentation of cardiac fibrosis was perivascular fibrosis that subsequently progressed to interstitial fibrosis.^41^ Meanwhile, perivascular fibrosis may reduce tissue availability to oxygen and nutrients and lead to a decline in coronary flow reserve and fractional flow reserve.^42^ Interestingly, a study supports our findings that PM_2.5_ exposure decreased coronary flow velocity which suggested perivascular fibrosis.^36^ Taken together, our data provide new insights that exposure to PM_2.5_ may contribute to the onset and development of cardiac remodeling. However, the mechanisms involved in PM_2.5_ related to the aggravation of cardiac fibrosis and dysfunction were unknown. In the process of cardiac fibrosis, cardiac fibroblasts are the major effector cells.^43^ Normal cardiac fibroblasts differentiation into contractile myofibroblasts and augment collagen production plays a key role in cardiac fibrosis.^44^ We confirmed that PM_2.5_ treatment promoted α‐SMA and collagen expression in cardiac fibroblasts, indicating that it involved in phenotypic modulation of fibroblasts into myofibroblasts (Figure 4). To our knowledge, our study provides the first evidence that PM_2.5_ could modulate the phenotype transformation of fibroblasts and contribute to cardiac fibrosis.

Over the past decade, accumulating evidence suggests that ROS and oxidative stress are involved in PM_2.5_‐mediated cardiovascular injury.^45^ The mitochondrion is a sensitive target of oxidative stress as well as environmental toxicants stimulus like PM_2.5_.^46^, ^47^ In contrast to the rather robust evidence base supporting the upregulation of ROS pathways in response to PM_2.5_ exposure, the regulation and expression of antioxidant defenses are relatively sparse.^48^, ^49^ There are a multitude of endogenous pathways that scavenge a range of ROS such as SOD2. SOD2 is an important member of the antioxidant enzyme family and presents in mitochondria which exerts a vital role in eliminating O_2_^•−^ and maintaining redox homeostasis.^50^, ^51^ It is clear that the activity of SOD2 can be regulated through post‐translational modifications.^52^ For instance, a reduction in SIRT3 decreases the deacetylation of SOD2, resulting in ROS accumulation.^53^, ^54^ SIRT3 is the most robust deacetylase and highly expressed in mitochondria that appears to play a crucial role on the regulation of oxidative stress and redox homeostasis.^55^ It has been demonstrated that SIRT3 plays a significant role in maintaining normal mitochondrial biological function via reversible protein acetylation.^56^, ^57^ In this study, we found PM_2.5_ exposure reduced the SIRT3 expression and activity, resulted in an increased acetylation of SOD2 and a loss in SOD2 activity. These changes lead to the accumulation of mitochondrial ROS and affect the redox homeostasis in mitochondria (Figures 3, 4, 5). Until recently, there have been several researches focused on the interactions between antioxidant genes and air pollution.^58^ These studies suggested that PM_2.5_ induced the change of antioxidant genes which performed important regulatory roles in the occurrence and development of cardiovascular diseases.

Melatonin is an endocrine substance mainly produced by the pineal gland. Interestingly, cardiac fibrosis could be detected in pinealectomized rats which indicating the latent association between melatonin with fibrosis accumulation.^59^ The protective effect of melatonin on cardiovascular injury in ApoE^−/−^ mice is probably a consequence of its antioxidant and anti‐inflammatory actions.^60^, ^61^, ^62^ Nardo et al recently reported that the beneficial effects of melatonin on ApoE^−/−^ mice by morphological and ^18^F‐fluorodeoxyglucose (^18^F‐FDG) positron emission tomography (PET) assessments, which revealed the protective role of melatonin on ApoE^−/−^ mice via suppressing macrophage polarization, reducing inflammation, preserving the activity of brown adipose tissue and improving the endothelial cell function.^63^ Meanwhile, it suggested that the application of ^18^F‐FDG PET/CT as a molecular imaging modality for early‐stage atherosclerosis may be an important means of early diagnosis and therapy. It has been identified as a potential new “gold standard” for early clinical diagnosis and grading regarding to its noninvasiveness. In our previous study, the ^18^F‐FDG PET/CT had been performed to explore the adverse effects of PM_2.5_ on pulmonary system.^64^ And the assessment method of ^18^F‐FDG PET/CT was recommended to explore the melatonin‐mediated protective role in future. Last but not least, the molecular mechanism reported in Nardo's study, such as the macrophage polarization, inflammation, and brown adipose tissue activity, may also contribute to our study on the observed protection of melatonin against PM_2.5_‐induced cardiac fibrosis. Therefore, more studies are encouraged to explore on this issue.

The role of melatonin in the protective effects of cardiac fibrosis induced by PM_2.5_ exposure in mice was also investigated in this study. As expected, melatonin alleviated cardiac remodeling in both myocyte hypertrophy and fibrosis during PM_2.5_ injury (Figures 1, 2, 3). Melatonin modulated the expression and activity of SIRT3 which altered the influence of PM_2.5_ exposure on oxidative stress of myocardial mitochondria. To gain a comprehensive understanding of melatonin against PM_2.5_‐triggered cardiac fibrosis, the human cardiac fibroblasts were further employed in our study. In line with results from the mice, our data demonstrated that melatonin effectively diminished the mitochondrial ROS level accompanied with the aid of upregulated SIRT3 to increase SOD2 expression and activity in PM_2.5_‐treated cardiac fibroblasts (Figure 6). To identify whether the SIRT3 played an important role in mediating the observed protective effects of melatonin after PM_2.5_ exposure, cardiac fibroblasts were then pretreated with 3‐TYP. The data documented that 3‐TYP pretreatment suppressed the rescue effects of melatonin on PM_2.5_‐induced fibroblast to myofibroblast phenoconversion (Figure 7). In addition, 3‐TYP significantly abolished the protective effect of melatonin via acetylated‐SOD2 expression and SOD2 inactivity resulted in an elevated level of mitochondrial ROS. Based on previous evidence, SIRT3‐deficient mice show increased acetylation and inhibition of several mitochondrial enzymes, exacerbated mitochondrial dysfunction.^65^ These data were consistent with melatonin suppressing mitochondrial ROS production and restoring cardiac fibroblasts phenotypic conversion through the upregulation of SIRT3 deacetylated SOD2 both in vitro and in vivo. A schematic diagram summarizing these results and mechanism is showing that melatonin effectively alleviates PM2.5‐induced cardiac dysfunction and fibrosis via inhibiting mitochondrial oxidative injury and regulating SIRT3‐mediated SOD2 deacetylation (Figure 8).

## 5 CONCLUSIONS

In summary, the present study demonstrates that PM_2.5_ could induce cardiac hypertrophy and fibrosis, particularly perivascular fibrosis. Additionally, melatonin administration could significantly reverse the PM_2.5_‐induced phenotypic modulation of cardiac fibroblasts into myofibroblasts. For the first time, our study finds that melatonin effectively alleviates PM_2.5_‐induced cardiac dysfunction and fibrosis via inhibiting mitochondrial oxidative injury and regulating SIRT3‐mediated SOD deacetylation. These results indicate that melatonin administration could be a prospective therapy for preventing and treating air pollution‐associated cardiac diseases.

## CONFLICT OF INTEREST

The authors declare that they have no conflicts of interest.

## AUTHOR CONTRIBUTION

Zhiwei Sun and Junchao Duan conceived and designed the experiments. Jinjin Jiang, Shuang Liang, Jingyi Zhang, Zhou Du, and Qing Xu performed the experiments. Jinjin Jiang, Shuang Liang, and Qing Xu analyzed the data. Zhiwei Sun and Junchao Duan contributed reagents/materials/analysis tools. Jinjin Jiang and Junchao Duan wrote the paper.

## References

[1] Shaddick G , Thomas ML , Amini H , et al. Data integration for the assessment of population exposure to ambient air pollution for global burden of disease assessment. Environ Sci Technol. 2018;52(16):9069‐9078. 29957991 10.1021/acs.est.8b02864

[2] Huang J , Pan X , Guo X , Li G . Health impact of China's Air Pollution Prevention and Control Action Plan: an analysis of national air quality monitoring and mortality data. Lancet Planet Health. 2018;2(7):e313‐e323. 30074894 10.1016/S2542-5196(18)30141-4

[3] Yue H , He C , Huang Q , Yin D , Bryan BA . Stronger policy required to substantially reduce deaths from PM pollution in China. Nat Commun. 2020;11(1):1462. 32193475 10.1038/s41467-020-15319-4 PMC7081205

[4] Wang Q , Wang J , Zhou J , Ban J , Li T . Estimation of PM‐associated disease burden in China in 2020 and 2030 using population and air quality scenarios: a modelling study. Lancet Planet Health. 2019;3(2):e71‐e80. 30797415 10.1016/S2542-5196(18)30277-8

[5] Pope CA , Burnett RT , Thurston GD , et al. Cardiovascular mortality and long‐term exposure to particulate air pollution: epidemiological evidence of general pathophysiological pathways of disease. Circulation. 2004;109(1):71‐77. 14676145 10.1161/01.CIR.0000108927.80044.7F

[6] Rajagopalan S , Al‐Kindi SG , Brook RD . Air pollution and cardiovascular disease: JACC state‐of‐the‐art review. J Am Coll Cardiol. 2018;72(17):2054‐2070. 30336830 10.1016/j.jacc.2018.07.099

[7] Kong P , Christia P , Frangogiannis NG . The pathogenesis of cardiac fibrosis. Cell Mol Life Sci. 2014;71(4):549‐574. 23649149 10.1007/s00018-013-1349-6 PMC3769482

[8] Ma Z , Yuan Y , Wu H , Zhang X , Tang Q . Cardiac fibrosis: new insights into the pathogenesis. Int J Biol Sci. 2018;14(12):1645‐1657. 30416379 10.7150/ijbs.28103 PMC6216032

[9] Frangogiannis NG . Cardiac fibrosis: cell biological mechanisms, molecular pathways and therapeutic opportunities. Mol Aspects Med. 2019;65:70‐99. 30056242 10.1016/j.mam.2018.07.001

[10] Aung N , Sanghvi MM , Zemrak F , et al. Association between ambient air pollution and cardiac morpho‐functional phenotypes: insights from the UK Biobank population imaging study. Circulation. 2018;138(20):2175‐2186. 30524134 10.1161/CIRCULATIONAHA.118.034856 PMC6250297

[11] Ge C , Hu L , Lou D , et al. Nrf2 deficiency aggravates PM‐induced cardiomyopathy by enhancing oxidative stress, fibrosis and inflammation via RIPK3‐regulated mitochondrial disorder. Aging. 2020;12(6):4836‐4865. 32182211 10.18632/aging.102906 PMC7138545

[12] Su X , Tian J , Li B , et al. Ambient PM2.5 caused cardiac dysfunction through FoxO1‐targeted cardiac hypertrophy and macrophage‐activated fibrosis in mice. Chemosphere. 2020;247:125881. 31978653 10.1016/j.chemosphere.2020.125881

[13] Jayasundara N . Ecological significance of mitochondrial toxicants. Toxicology. 2017;391:64‐74. 28778750 10.1016/j.tox.2017.07.015

[14] Janssen BG , Munters E , Pieters N , et al. Placental mitochondrial DNA content and particulate air pollution during in utero life. Environ Health Perspect. 2012;120(9):1346‐1352. 22626541 10.1289/ehp.1104458 PMC3440109

[15] Hou L , Zhu Z , Zhang X , et al. Airborne particulate matter and mitochondrial damage: a cross‐sectional study. Environ Health. 2010;9:48. 20696069 10.1186/1476-069X-9-48 PMC2928195

[16] Xia T , Korge P , Weiss JN , et al. Quinones and aromatic chemical compounds in particulate matter induce mitochondrial dysfunction: implications for ultrafine particle toxicity. Environ Health Perspect. 2004;112(14):1347‐1358. 15471724 10.1289/ehp.7167 PMC1247559

[17] Torrealba N , Aranguiz P , Alonso C , Rothermel BA , Lavandero S . Mitochondria in structural and functional cardiac remodeling. Adv Exp Med Biol. 2017;982:277‐306. 28551793 10.1007/978-3-319-55330-6_15

[18] Kim HK , Nilius B , Kim N , Ko KS , Rhee BD , Han J . Cardiac response to oxidative stress induced by mitochondrial dysfunction. Rev Physiol Biochem Pharmacol. 2016;170:101‐127. 26744346 10.1007/112_2015_5004

[19] Bai P , Canto C , Brunyánszki A , et al. PARP‐2 regulates SIRT1 expression and whole‐body energy expenditure. Cell Metab. 2011;13(4):450‐460. 21459329 10.1016/j.cmet.2011.03.013 PMC3108571

[20] Houtkooper RH , Pirinen E , Auwerx J . Sirtuins as regulators of metabolism and healthspan. Nat Rev Mol Cell Biol. 2012;13(4):225‐238. 22395773 10.1038/nrm3293 PMC4872805

[21] Jokinen R , Pirnes‐Karhu S , Pietiläinen KH , Pirinen E . Adipose tissue NAD‐homeostasis, sirtuins and poly(ADP‐ribose) polymerases ‐important players in mitochondrial metabolism and metabolic health. Redox Biol. 2017;12:246‐263. 28279944 10.1016/j.redox.2017.02.011 PMC5343002

[22] Cantó C , Sauve AA , Bai P . Crosstalk between poly(ADP‐ribose) polymerase and sirtuin enzymes. Mol Aspects Med. 2013;34(6):1168‐1201. 23357756 10.1016/j.mam.2013.01.004 PMC3676863

[23] Dikalova AE , Pandey A , Xiao L , et al. Mitochondrial deacetylase Sirt3 reduces vascular dysfunction and hypertension while Sirt3 depletion in essential hypertension is linked to vascular inflammation and oxidative stress. Circ Res. 2020;126(4):439‐452. 31852393 10.1161/CIRCRESAHA.119.315767 PMC7035170

[24] Tordjman S , Chokron S , Delorme R , et al. Melatonin: pharmacology, functions and therapeutic benefits. Curr Neuropharmacol. 2017;15(3):434‐443. 28503116 10.2174/1570159X14666161228122115 PMC5405617

[25] Opie LH , Lecour S . Melatonin has multiorgan effects. Eur Heart J Cardiovasc Pharmacother. 2016;2(4):258‐265. 27533945 10.1093/ehjcvp/pvv037

[26] Ji Z , Wang Z , Chen Z , et al. Melatonin attenuates chronic cough mediated by oxidative stress via transient receptor potential melastatin‐2 in guinea pigs exposed to particulate matter 2.5. Physiol Res. 2018;67(2):293‐305. 29303602 10.33549/physiolres.933654

[27] Lee F‐Y , Lee MS , Wallace CG , et al. Short‐interval exposure to ambient fine particulate matter (PM2.5) exacerbates the susceptibility of pulmonary damage in setting of lung ischemia‐reperfusion injury in rodent: pharmacomodulation of melatonin. Biomed Pharmacother. 2019;113:108737. 30852418 10.1016/j.biopha.2019.108737

[28] Nelson RH . Hyperlipidemia as a risk factor for cardiovascular disease. Prim Care. 2013;40(1):195‐211. 23402469 10.1016/j.pop.2012.11.003 PMC3572442

[29] Yang B‐Y , Guo Y , Markevych I , et al. Association of long‐term exposure to ambient air pollutants with risk factors for cardiovascular disease in China. JAMA Netw Open. 2019;2(3):e190318. 30848806 10.1001/jamanetworkopen.2019.0318 PMC6484675

[30] Pan H , Cheung S , Chen F , et al. Short‐term effects of ambient air pollution on ST‐elevation myocardial infarction events: are there potentially susceptible groups? Int J Environ Res Public Health. 2019;16(19):3760. 10.3390/ijerph16193760 PMC6801768 31591299

[31] Li N , Chen G , Liu F , et al. Associations between long‐term exposure to air pollution and blood pressure and effect modifications by behavioral factors. Environ Res. 2020;182:109109. 32069739 10.1016/j.envres.2019.109109 PMC7043011

[32] Liu J , Liang S , Du Z , et al. PM2.5 aggravates the lipid accumulation, mitochondrial damage and apoptosis in macrophage foam cells. Environ Pollut. 2019;249:482‐490. 30928520 10.1016/j.envpol.2019.03.045

[33] Jiang J , Li Y , Liang S , et al. Combined exposure of fine particulate matter and high‐fat diet aggravate the cardiac fibrosis in C57BL/6J mice. J Hazard Mater. 2020;391:122203. 32171159 10.1016/j.jhazmat.2020.122203

[34] Mannucci PM , Harari S , Franchini M . Novel evidence for a greater burden of ambient air pollution on cardiovascular disease. Haematologica. 2019;104(12):2349‐2357. 31672903 10.3324/haematol.2019.225086 PMC6959193

[35] Scherrer‐Crosbie M , Kurtz B . Ventricular remodeling and function: insights using murine echocardiography. J Mol Cell Cardiol. 2010;48(3):512‐517. 19615377 10.1016/j.yjmcc.2009.07.004 PMC2823993

[36] Wold LE , Ying Z , Hutchinson KR , et al. Cardiovascular remodeling in response to long‐term exposure to fine particulate matter air pollution. Circ Heart Fail. 2012;5(4):452‐461. 22661498 10.1161/CIRCHEARTFAILURE.112.966580 PMC3617499

[37] Zhang Y , Ji X , Ku T , Li B , Li G , Sang N . Ambient fine particulate matter exposure induces cardiac functional injury and metabolite alterations in middle‐aged female mice. Environ Pollut. 1987;2019(248):121‐132. 10.1016/j.envpol.2019.01.080 30784831

[38] Ponikowski P , Voors AA , Anker SD , et al. 2016 ESC Guidelines for the diagnosis and treatment of acute and chronic heart failure: the Task Force for the diagnosis and treatment of acute and chronic heart failure of the European Society of Cardiology (ESC) developed with the special contribution of the Heart Failure Association (HFA) of the ESC. Eur Heart J. 2016;37(27):2129‐2200. 27206819 10.1093/eurheartj/ehw128

[39] Seferovic PM , Ponikowski P , Anker SD , et al. Clinical practice update on heart failure 2019: pharmacotherapy, procedures, devices and patient management. An expert consensus meeting report of the Heart Failure Association of the European Society of Cardiology. Eur J Heart Fail. 2019;21(10):1169‐1186. 31129923 10.1002/ejhf.1531

[40] Wu Q , Xiao Y , Yuan Y , et al. Mechanisms contributing to cardiac remodelling. Clin Sci. 2017;131(18):2319‐2345. 10.1042/CS20171167 28842527

[41] Kuwahara F , Kai H , Tokuda K , et al. Hypertensive myocardial fibrosis and diastolic dysfunction. Hypertension. 2004;43(4):739‐745. 14967845 10.1161/01.HYP.0000118584.33350.7d

[42] Kai H , Mori T , Tokuda K , et al. Pressure overload‐induced transient oxidative stress mediates perivascular inflammation and cardiac fibrosis through angiotensin II. Hypertens Res. 2006;29(9):711‐718. 17249527 10.1291/hypres.29.711

[43] Travers JG , Kamal FA , Robbins J , Yutzey KE , Blaxall BC . Cardiac fibrosis: the fibroblast awakens. Circ Res. 2016;118(6):1021‐1040. 26987915 10.1161/CIRCRESAHA.115.306565 PMC4800485

[44] Ivey MJ , Tallquist MD . Defining the Cardiac Fibroblast. Circ J. 2016;80(11):2269‐2276. 27746422 10.1253/circj.CJ-16-1003 PMC5588900

[45] Mudway IS , Kelly FJ , Holgate ST . Oxidative stress in air pollution research. Free Radic Biol Med. 2020;151:2‐6. 32360613 10.1016/j.freeradbiomed.2020.04.031 PMC7252129

[46] Meyer JN , Leung MCK , Rooney JP , et al. Mitochondria as a target of environmental toxicants. Toxicol Sci. 2013;134(1):1‐17. 23629515 10.1093/toxsci/kft102 PMC3693132

[47] Caito SW , Aschner M . Mitochondrial redox dysfunction and environmental exposures. Antioxid Redox Signal. 2015;23(6):578‐595. 25826672 10.1089/ars.2015.6289 PMC4544749

[48] Rao X , Zhong J , Brook RD , Rajagopalan S . Effect of particulate matter air pollution on cardiovascular oxidative stress pathways. Antioxid Redox Signal. 2018;28(9):797‐818. 29084451 10.1089/ars.2017.7394 PMC5831906

[49] Kelly FJ , Fussell JC . Role of oxidative stress in cardiovascular disease outcomes following exposure to ambient air pollution. Free Radic Biol Med. 2017;110:345‐367. 28669628 10.1016/j.freeradbiomed.2017.06.019

[50] Zou X , Ratti BA , O'Brien JG , et al. Manganese superoxide dismutase (SOD2): is there a center in the universe of mitochondrial redox signaling? J Bioenerg Biomembr. 2017;49(4):325‐333. 28616679 10.1007/s10863-017-9718-8

[51] Brand MD . Mitochondrial generation of superoxide and hydrogen peroxide as the source of mitochondrial redox signaling. Free Radic Biol Med. 2016;100:14‐31. 27085844 10.1016/j.freeradbiomed.2016.04.001

[52] Palma FR , He C , Danes JM , et al. Mitochondrial superoxide dismutase: what the established, the intriguing, and the novel reveal about a key cellular redox switch. Antioxid Redox Signal. 2020;32(10):701‐714. 31968997 10.1089/ars.2019.7962 PMC7047081

[53] Tao R , Coleman MC , Pennington JD , et al. Sirt3‐mediated deacetylation of evolutionarily conserved lysine 122 regulates MnSOD activity in response to stress. Mol Cell. 2010;40(6):893‐904. 21172655 10.1016/j.molcel.2010.12.013 PMC3266626

[54] Wu J , Zeng Z , Zhang W , et al. Emerging role of SIRT3 in mitochondrial dysfunction and cardiovascular diseases. Free Radical Res. 2019;53(2):139‐149. 30458637 10.1080/10715762.2018.1549732

[55] Dikalov SI , Dikalova AE . Crosstalk between mitochondrial hyperacetylation and oxidative stress in vascular dysfunction and hypertension. Antioxid Redox Signal. 2019;31(10):710‐721. 30618267 10.1089/ars.2018.7632 PMC6708267

[56] Parodi‐Rullán RM , Chapa‐Dubocq XR , Javadov S . Acetylation of mitochondrial proteins in the heart: the role of SIRT3. Front Physiol. 2018;9:1094. 30131726 10.3389/fphys.2018.01094 PMC6090200

[57] Kane AE , Sinclair DA . Sirtuins and NAD in the development and treatment of metabolic and cardiovascular diseases. Circ Res. 2018;123(7):868‐885. 30355082 10.1161/CIRCRESAHA.118.312498 PMC6206880

[58] Fuertes E , van der Plaat DA , Minelli C . Antioxidant genes and susceptibility to air pollution for respiratory and cardiovascular health. Free Radic Biol Med. 2020;151:88‐98. 32007521 10.1016/j.freeradbiomed.2020.01.181

[59] Mizrak B , Parlakpinar H , Acet A , Turkoz Y . Effects of pinealectomy and exogenous melatonin on rat hearts. Acta Histochem. 2004;106(1):29‐36. 15032326 10.1016/j.acthis.2003.10.003

[60] Li H , Li J , Jiang X , et al. Melatonin enhances atherosclerotic plaque stability by inducing prolyl‐4‐hydroxylase α1 expression. J Hypertens. 2019;37(5):964‐971. 30335670 10.1097/HJH.0000000000001979

[61] Ding S , Lin N , Sheng X , et al. Melatonin stabilizes rupture‐prone vulnerable plaques via regulating macrophage polarization in a nuclear circadian receptor RORα‐dependent manner. J Pineal Res. 2019;67(2):e12581. 31009101 10.1111/jpi.12581

[62] Zhang Y , Liu X , Bai X , et al. Melatonin prevents endothelial cell pyroptosis via regulation of long noncoding RNA MEG3/miR‐223/NLRP3 axis. J Pineal Res. 2018;64(2):e12449. 10.1111/jpi.12449 29024030

[63] Nardo L , Rezzani R , Facchetti L , et al. Beneficial effects of melatonin on apolipoprotein‐E knockout mice by morphological and F‐FDG PET/CT assessments. Int J Mol Sci. 2020;21(8):2920. 10.3390/ijms21082920 PMC7216051 32331251

[64] Sun B , Shi Y , Li Y , et al. Short‐term PM exposure induces sustained pulmonary fibrosis development during post‐exposure period in rats. J Hazard Mater. 2020;385:121566. 31761645 10.1016/j.jhazmat.2019.121566

[65] Paulin R , Dromparis P , Sutendra G , et al. Sirtuin 3 deficiency is associated with inhibited mitochondrial function and pulmonary arterial hypertension in rodents and humans. Cell Metab. 2014;20(5):827‐839. 25284742 10.1016/j.cmet.2014.08.011

